# *Cryptosporidium* within a One health framework: a comprehensive review of public health impact, environmental concerns, and emerging strategies for prevention and treatment

**DOI:** 10.3389/fvets.2026.1731158

**Published:** 2026-03-12

**Authors:** Refaat Ras, Adel Abdelkhalek, Enrique Raya-Álvarez, Rawan Muhammad Shady, Qwait AlGabbani, Abdelbaset E. Abdelbaset, Ali S. A. Saleem, Mustafa Shukry, Ahmed Agil, Ehab Kotb Elmahallawy

**Affiliations:** 1 Department of Microbiology and Parasitology, Faculty of Veterinary Medicine, Badr University in Cairo (BUC), Badr City, Egypt; 2 Department of Parasitology, Faculty of Veterinary Medicine, Zagazig University, Zagazig, Egypt; 3 Department of Food Hygiene, Safety and Technology, Faculty of Veterinary Medicine, Badr University in Cairo (BUC), Badr City, Egypt; 4 Rheumatology Department, Hospital Universitario San Cecilio, Granada, Spain; 5 Biotechnology Department, Faculty of Science, Cairo University, Giza, Egypt; 6 Department of Biology, College of Sciences and Humanities, Prince Sattam Bin Abdulaziz University, Al-Kharj, Saudi Arabia; 7 Laboratory of Parasitology, Graduate School of Infectious Diseases, Faculty of Veterinary Medicine, Hokkaido University, Sapporo, Japan; 8 Department of Clinical Pathology, Faculty of Veterinary Medicine, Assiut University, Assiut, Egypt; 9 Animal Production Department, Faculty of Agriculture, Sohag University, Sohag, Egypt; 10 Department of Biomedical Sciences, College of Veterinary Medicine, King Faisal University, Al-Ahsa, Saudi Arabia; 11 Department of Pharmacology, Biohealth Institute Granada (IBs Granada) and Neuroscience Institute, School of Medicine, University of Granada, Granada, Spain; 12 Departamento de Sanidad Animal, Grupo de Investigación en Sanidad Animal y Zoonosis (GISAZ), Universidad de Córdoba, Córdoba, Spain; 13 Department of Zoonoses, Faculty of Veterinary Medicine, Sohag University, Sohag, Egypt

**Keywords:** *Cryptosporidium*, environment, One health, prevention, public health, treatment, zoonoses

## Abstract

*Cryptosporidium* species are globally distributed parasites and are a major cause of cryptosporidiosis, a diarrheal disease that disproportionately affects immunocompromised individuals and young children in low-resource settings. *Cryptosporidium* is widely regarded as a critical contaminant of drinking water and is strongly associated with an increased risk of waterborne disease, posing a serious threat to public health. Furthermore, agricultural environments can serve as sources of contamination with *Cryptosporidium* oocysts through fecal material originating from both humans and animals. Despite their major zoonotic relevance, critical gaps remain in understanding their true public health burden, transmission pathways, and ways to effectively translate emerging knowledge into prevention and treatment strategies. Currently, nitazoxanide is the only FDA-approved treatment for cryptosporidiosis; however, its efficacy in vulnerable populations remains limited. Safe and effective chemotherapeutics are urgently needed; yet drug evaluation methods lack standardization, and no reliable vaccine surrogates capable of inducing protective immunity have been identified. Additionally, the identification of vaccination surrogates that confer protective immune responses against *Cryptosporidium* infection remains an unresolved need. In addition, *Cryptosporidium* diagnosis relies on fecal microscopy, antigen-based assays for rapid detection, and molecular methods, such as PCR, for sensitive species identification and genotyping. Emerging approaches, including next-generation sequencing, promise improved multiplex detection and epidemiological resolution but require further integration into routine clinical laboratory workflows. This review summarizes the advances in *Cryptosporidium* therapeutics, including herbal, chemical, and pharmaceutical approaches, as well as chemical agents utilized for water treatment and environmental control, highlighting associated safety concerns and diagnostics. It further outlines priority areas for future research, such as the development of novel drug candidates, host-targeted therapies, and next-generation vaccine platforms. Finally, it underscores the complex, multifactorial challenges of controlling *Cryptosporidium* and calls for a One Health approach that integrates human, animal, and environmental health to mitigate its global impact.

## 1 Background

*Cryptosporidium* is an apicomplexan parasite that colonizes the gastrointestinal tract of humans and animals, causing cryptosporidiosis, a disease characterized by diarrhea, abdominal pain, and severe dehydration (1). Although often self-limiting, the infection is especially severe in immunocompromised individuals, including people living with HIV/AIDS, where it often progresses to chronic and debilitating illness (2). As a leading waterborne pathogen, *Cryptosporidium* imposes a substantial disease burden across both developed and developing countries (3). The burden of the disease is most pronounced in settings with inadequate sanitation and limited access to clean water, placing young children, the elderly, and immunocompromised populations at highest risk. Moreover, the Global Enteric Multicenter Study identified *Cryptosporidium* as one of the four leading pathogens, causing moderate to severe diarrhea in children under 5 years of age in sub-Saharan Africa and South Asia (2). In developed countries, outbreaks are often associated with recreational water sources like swimming pools and water parks, highlighting the persistence of *Cryptosporidium* spp. as a public health concern (4). Millions of cases are recorded worldwide each year, underscoring the urgent need for more effective prevention and treatment strategies (5). The parasite’s remarkable resilience stems from its tough, thick-walled oocysts, which enable it to survive harsh environmental conditions and resist many common water disinfectants. This resilience has contributed to an increase in the number of outbreaks worldwide (6).

Transmission of *Cryptosporidium* spp. occurs predominantly through the fecal-oral route, with humans becoming infected primarily through ingestion of oocysts present in contaminated water, food, or surfaces. The species most commonly causing human disease are *C. parvum* and *C. hominis*, which together account for the vast majority of infections globally (7). Notably, certain species of *Cryptosporidium*, particularly *C. parvum*, infecting a wide range of hosts including livestock, companion animals, and rodents, which serve as major reservoirs, shedding large numbers of oocysts into the environment and contaminating water, food, and surfaces (8–11). Moreover, wild birds can also contribute to oocyst contamination of water and food supplies, further complicating the transmission dynamics (12). A recent report indicated that the global prevalence of *Cryptosporidium* in wild birds was estimated to be 3.96% (1945 of 49,129), encompassing six species (*C. parvum, C. meleagridis, C. andersoni, C. avium, C. galli,* and *C. baileyi*) and five distinct genotypes (Goose genotype I, Goose genotype II, Avian genotype I, Avian genotype III, and Avian genotype VI) (13). Furthermore, the detection of *C. bovis* and *C. parvum* in yak animals highlights cryptosporidiosis as a One Health concern, reflecting risks at the animal–human–environment interface which support the need for integrated prevention and control strategies to protect livestock health and reduce potential public health impact (14). Other *Cryptosporidium* species, including *C. andersoni*, *C. felis*, *C. meleagridis*, *C. suis* and *C. canis* have been identified in both animals and humans (9). In addition, *C. ubiquitum*, *C. viatorum*, and *C. cuniculus*, along with various other genotypes, can also infect humans (15, 16).

**Figure 1:**
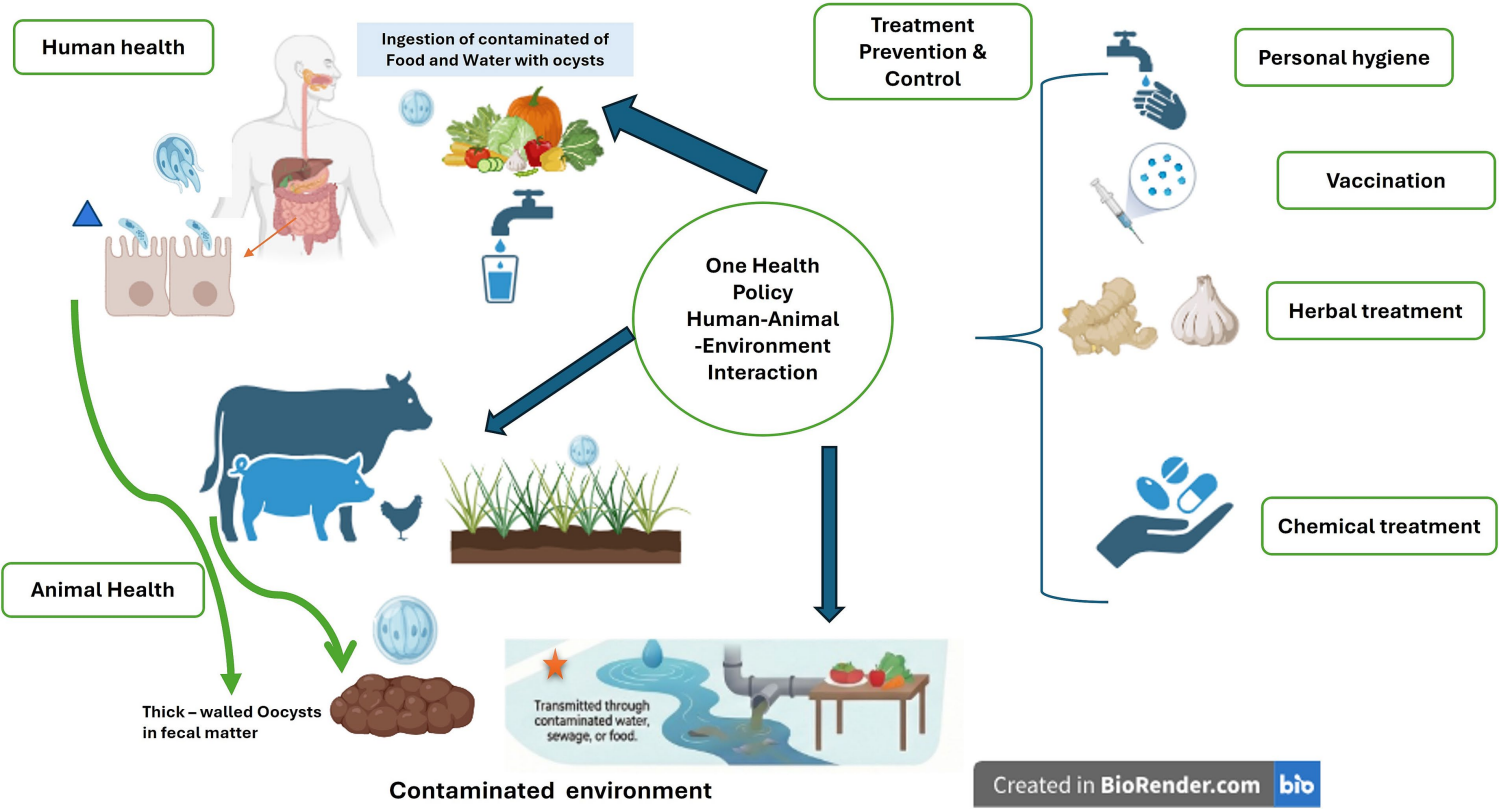
Cryptosporidiosis and One Health: interactions between human, animal, and environmental health. The image was generated through Biorender software (Free trial version). Blue triangle symbol (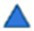), excysted *Cryptosporidium* oocysts with invasive sporozoite, other stages of schizogony are and gamatogony not demonstrated. Red asterisk symbol (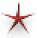), the image of environment was generated by Notebook software.

Taken together, the zoonotic nature of the parasite underscores the urgent need for coordinated One Health approaches, involving veterinary, medical, and environmental sectors, to monitor and mitigate transmission risks. Clearly, precise characterization of *Cryptosporidium* genetic diversity is critical for source tracking in both animals and humans (17, 18). Notably, the actual burden of cryptosporidiosis in both human and veterinary health remains unclear, as many cases of infection are often undiagnosed or unreported. Therefore, effective control strategies require an integrated approach, including developing vaccines for livestock and humans, improving sanitation, implementing better risk management, and employing advanced genotyping to track and differentiate *Cryptosporidium* species. Treating livestock and human waste to reduce oocyst viability is crucial to minimizing environmental contamination and protecting water catchments. Public health education on transmission routes and preventative measures, such as handwashing, are also essential for reducing disease incidence. Collectively, a successful One Health strategy not only reduces disease in livestock but also improves water quality, preserves biodiversity, and lowers the risk to human populations, as illustrated in Figure 1, which highlights the interconnected benefits of this integrated approach.

## 2 Literature search

A comprehensive search was conducted in PubMed, SCOPUS, Google Scholar, ScienceDirect and other databases to find relevant research articles without time limitation up to 04 December 2025 using the following criteria and keywords; “*Cryptosporidium*” and “Cryptosporidiosis” and “One Health” and “treatment” and “Therapies” and “Herbal” and “environmental” and “Public Health” and “Vaccination” and “Drug resistance” and Diagnostics. Articles related to *Cryptosporidium* are included in the list of references. Overall, the obtained articles were found to be relevant to the subject of the current study, and valuable scientific knowledge was summarized in the following sections.

## 3 Pathogenesis and clinical manifestations

As depicted in Figure 1, *Cryptosporidium* oocysts are shed in the feces of infected humans and animals and contaminate water, soil, fresh produce, and food-processing environments. Their strong resistance to chlorine and ability to survive for months in moist conditions make them a persistent environmental hazard (19). Infection begins when a person or animal consumes *Cryptosporidium* oocysts through contaminated water or food. Each oocyst carries four sporozoites that are released in the small intestine. These sporozoites then invade the lining of the intestine, triggering an immune response that leads to inflammation and damage to the intestinal barrier, resulting in diarrhea and nutrient malabsorption (15, 20). In addition, animals particularly calves, lambs, goats, and young companion animals serve as major sources of oocyst contamination (10), increasing the environmental load and raising the risk of zoonotic transmission to humans, especially farmers, veterinarians, and children living near livestock facilities (9, 21). The severity of illness depends largely on the strength of the host’s immune system. In healthy individuals, the infection often causes mild to moderate diarrhea, along with abdominal discomfort, nausea, and sometimes vomiting, including subclinical infections, wherein symptoms may be less evident (22). Most people recover on their own within a couple of weeks (1). However, for immunocompromised patients, such as those living with HIV/AIDS, elderly, or malnourished children, the infection can cause severe, persistent diarrhea, significant weight loss, and life-threatening dehydration (15). Extra-intestinal manifestations, such as biliary tract infections, are also more common in these vulnerable groups. Furthermore, a previous study (23) demonstrated that *Cryptosporidium* infection is associated with distinct alterations in the fungal community of pigs, underscoring the interconnected roles of host, pathogen, and microbiome which influences host immune response and gut homeostasis. Given its transmission and pathogenicity, understanding how *Cryptosporidium* interacts with the host and triggers immune responses, as well as the spectrum of symptoms it produces, is critical for developing more effective treatments and preventive strategies (16).

## 4 Current treatment approaches

### 4.1 Overview of currently approved treatments

Current treatment approaches for cryptosporidiosis remain limited, with nitazoxanide being the only FDA-approved drug specifically indicated for this infection (24). Other agents, such as paromomycin and azithromycin, have been used alongside supportive care to help control symptoms and improve outcomes in certain patients (25). However, their inconsistent effectiveness and lack of a fully dependable cure highlights the urgent need for new, more effective therapies (26). The following sections outline the existing treatment strategies, highlighting their active components and mechanisms of action (Table 1).

**Table 1:** Integrated summary of preclinical and clinical data on therapeutic efficacy against *Cryptosporidium* infection.

Treatment	Category	Pros	Cons	Gaps and future direction	References
Nitazoxanide	FDA-approved	Effective in immunocompetent individuals	Reduced efficacy in immunocompromised patients	Optimization of dosing and combination therapies	(24, 31)
Paromomycin	Off-label use	Inhibits protein synthesis in parasites	Nephrotoxicity	Lack of FDA approval, need for clinical trials	(30, 35)
Azithromycin	Off-label use	Broad-spectrum activity	Variable efficacy in immunocompromised patients	Clarification of optimal dosing and combination with other agents	(38)
Benzoxaboroles	Inhibits leucyl-tRNA synthetase	Potent against drug-resistant strains	Limited clinical data	Further clinical trials to establish safety and efficacy	(42)
PI(4)K inhibitors	Disrupts phosphoinositide metabolism	Novel Mechanism of action	Limited clinical validation	Development of formulations suitable for clinical use	(44)
Protease inhibitors	Inhibits essential proteases of the parasite	Potential for broad-spectrum activity	Limited efficacy data in clinical settings	Optimization of compound potency and pharmacokinetic profiles	(115)
Macrolides (e.g., Clarithromycin)	Inhibits protein synthesis	Alternative to azithromycin	Limited clinical data	Comparative trials with azithromycin for efficacy and safety	(116)
Rifabutin	Inhibits bacterial RNA polymerase	Potential for anti-*Cryptosporidium* activity	Potential drug interactions with other medications	Optimization of dosing regimens and evaluation in clinical trials	(117)
Highly active antiretroviral therapy (HAART)	Suppresses HIV replication	Manages underlying HIV infection	Potential for drug interactions and side effects	Optimization of regimens for dual management of HIV and *Cryptosporidium*	(118)
Supportive therapy*	Symptomatic relief and hydration	Improves patient comfort and hydration	Does not eliminate *Cryptosporidium* infection	Integration with specific anti-*Cryptosporidium* treatments	Supportive care guidelines and clinical practice

*Supportive therapy including fluid and electrolyte administration in case of dehydration and diarrhea.

#### 4.1.1 Nitazoxanide

Nitazoxanide is widely recognized as an important option for managing cryptosporidiosis because it acts directly on the energy metabolism of *Cryptosporidium* parasites. With FDA approval for this particular indication, nitazoxanide has demonstrated its efficacy in reducing the severity and duration of diarrhea associated with *Cryptosporidium* infection in both immunocompetent and immunocompromised individuals (27). Its antiparasitic activity is mediated through inhibition of the enzyme pyruvate:ferredoxin oxidoreductase, a key component of the parasite’s anaerobic energy metabolism, thereby impairing *Cryptosporidium* survival and replication (24). However, its therapeutic success is more consistent in individuals with intact immunity, whereas outcomes are often suboptimal in severely immunocompromised populations. Therefore, Elmansory *et al.* 2025 (28) conducted an evaluation of the efficacy of ivermectin (IVM) and albendazole (ALB), both individually and in combination with nitazoxanide (NTZ), in immunosuppressed mice infected with *C. parvum*. The combination therapies, particularly NTZ in conjunction with IVM, resulted in the most significant reductions in oocyst shedding, followed by NTZ combined with ALB. These combinations proved superior to all monotherapies, underscoring the limited effectiveness of NTZ alone in immunocompromised hosts. Furthermore, a randomized controlled trial study by Amadi et al. (29), examined 50 children with HIV and 50 without HIV. Among the HIV-negative children, diarrhea resolved in 14 of 25 who received nitazoxanide, compared to 5 of 22 who were given a placebo, showing a 33% difference. *C. parvum* was cleared in 13 of the 25 children treated with nitazoxanide, while only 3 of the 22 children in the placebo group experienced clearance (difference of 38%). By the eighth day, four children (18%) in the placebo group had died, whereas there were no deaths in the nitazoxanide group. However, nitazoxanide demonstrated no therapeutic efficacy in HIV-seropositive children, and no significant adverse effects were observed in either treatment group.

The drug is generally well tolerated, with mild gastrointestinal disturbances such as nausea and abdominal discomfort being the most commonly reported side effects (30, 31). Although nitazoxanide is the only approved antiparasitic drug for use against *Cryptosporidium* in humans, its effectiveness in veterinary species is not well established (32, 33).

#### 4.1.2 Paromomycin

Paromomycin is an aminoglycoside antibiotic used in the treatment of cryptosporidiosis, particularly among immunocompromised patients for whom therapeutic alternatives remain limited (34). Its mode of action involves binding to the 30S ribosomal subunit of the parasite, thereby disrupting protein synthesis and inhibiting parasite replication, which supports the clearance of the infection from the gastrointestinal tract (35). Although paromomycin showed moderate efficacy, it has not received specific approval from the FDA for cryptosporidiosis, which limits its widespread clinical application. In addition, paromomycin had low toxicity and moderate therapeutic efficacy against cryptosporidiosis at high doses in an animal model system (36). A recent study indicated that paromomycin, now available in the UK through veterinary prescription, reduces oocyst shedding and alleviates diarrhea; however, it also poses a risk of toxicity (8). Owing to these limitations, paromomycin is frequently administered in combination with other therapeutic agents to improve treatment success and reduce side effects, emphasizing the urgent need for the development of new and more effective therapies against cryptosporidiosis (37).

#### 4.1.3 Azithromycin

Azithromycin, a macrolide antibiotic, has been evaluated as a therapeutic option for cryptosporidiosis, especially in immuno-compromised individuals such as those living with HIV/AIDS. Its mechanism involves binding to the 50S ribosomal subunit of the parasite and inhibits protein synthesis in which impairs parasite replication, which supports clearance from the intestinal tract (38). Although azithromycin demonstrates some antiparasitic activity, its efficacy as monotherapy is limited. It is therefore most often administered in combination with other agents to improve treatment outcomes (25). Reported adverse effects include gastrointestinal disturbances and potential drug–drug interaction. Careful clinical monitoring is required to ensure patient safety, particularly in patients with pre-existing liver dysfunction or those receiving concurrent therapies (25).

#### 4.1.4 Halofuginone lactate

Halofuginone lactate (HFL), a synthetic quinazolinone compound, exhibits cryptosporidiostatic activity that targets the sporozoite and merozoite stages of *C. parvum*. It is used for both therapeutic and prophylactic purposes, effectively delaying the onset of infection, reducing oocyst shedding, and mitigating the clinical severity of cryptosporidiosis in calves (39). A previous research (40) found that halofuginone lactate and paromomycin are effective treatments for cryptosporidiosis in calves, and recommended their use to manage *C. parvum* infections. Furthermore, HFL is currently the only approved treatment for *Cryptosporidium* infection in calves. It is licensed for use in regions such as the United Kingdom and the European Union, but has not yet received approval in the United States (41).

### 4.2 Novel drug candidates

Recent efforts to develop new therapies for cryptosporidiosis have focused on exploiting the parasite-specific metabolic pathways and essential enzymatic targets. Benzoxaboroles have emerged as promising candidates through to their inhibition of leucyl tRNA synthetase, an enzyme indispensable for protein synthesis in *Cryptosporidium* spp. (42, 43). Preclinical studies have demonstrated that benzoxaboroles exhibit potent activity against both drug sensitive and resistant strains, highlighting their potential as next-generation antiparasitic agents. Consequently, the 6-carboxamide benzoxaborole AN7973 may possess therapeutic potential for a diverse range of patient populations affected by cryptosporidiosis, including individuals with AIDS, transplant recipients, and malnourished children. In addition to demonstrating efficacy in murine models of both acute and chronic infections, AN7973 reduced *C. parvum* fecal shedding, diarrhea, and dehydration in a neonatal calf model that closely mimics the disease observed in human infants (43).

Another target of considerable interest is phosphatidylinositol 4-kinase (PI4K), an enzyme that plays a central role in phosphoinositide metabolism and is vital for the survival and intracellular development of the parasite (44).

*Plasmodium* PI(4)K inhibitors diaryl-aminopyridine (MMV390048), have demonstrated substantial suppression of *C. parvum* proliferation in both *in vitro* assays and murine infection models, with minimal adverse effects. Similarly, compounds such as KDU73 and BQR695 have shown comparable efficacy, achieving significant reductions in parasite burden in preclinical evaluations (45). Moreover, Manjunatha *et al.* 2017 (44) highlighted the potential of the pyrazolopyridine compound KDU731 as a promising therapeutic candidate against *C. parvum* and *C. hominis*. In contrast to nitazoxanide, KDU731 demonstrated significant *in vivo* efficacy in immunocompromised murine models. Furthermore, when administered to neonatal calves, a model that closely replicates the treatment challenges encountered in young, malnourished children; it resulted in a marked reduction in parasite shedding and a rapid amelioration of diarrhea and dehydration.

Collectively, these advances underscore an active pursuit of novel compounds that could complement or replace current therapies, providing much needed alternatives for high-risk groups who face severe forms of this infection (46, 47).

### 4.3 Host-targeted therapies

Current investigations into host-directed treatments for *Cryptosporidium* aim to leverage the host immune machinery to suppress parasite development and limit disease progression. A key approach involves the use of immunostimulatory agents designed to activate innate immune responses and enhance the clearance of infected cells. Agents such as specific cytokines and interferons have shown promise in boosting immune reactivity against *Cryptosporidium* species (48). Another promising approach focuses on blocking the cellular pathways or surface receptors that the parasite relies on to invade and persist within the intestinal epithelium. Targeting host factors rather than the parasite directly offers the advantage of lowering the potential for drug resistance. For example, Potiron et al. (49) demonstrated that modulating dendritic cell responses in experimental models reduced both parasite burden and disease intensity. Continued efforts are directed at identifying new host factors and refining immunotherapeutic interventions to offer additional options for managing cryptosporidiosis, especially in patients with compromised immunity (49, 50).

### 4.4 Herbal remedies

Herbal medicines are gaining attention as promising candidates in the fight against *Cryptosporidium* infections due to their natural origin and long-standing use in traditional healing. The following subsections summarize the most studied herbal candidates and the mechanisms underlying their effects.

#### 4.4.1 Garlic (*Allium sativum*)

Garlic (*Allium sativum*) has been investigated for its potential to treat *Cryptosporidium* due to its well-documented antimicrobial properties. The active component of garlic, allicin, has shown effectiveness in inhibiting the growth of *C. parvum in vivo*, disrupting the parasite’s metabolic processes (51). Studies indicated that allicin interferes with thiol-dependent enzymatic systems within the parasite, impeding its ability to replicate and survive (52, 53). Furthermore, garlic’s immunomodulatory effects may bolster the host’s immune response, offering a dual mechanism of action against the infection (54).

#### 4.4.2 Berberis vulgaris

*Berberis vulgaris* (commonly known as barberry) shows considerable therapeutic potential against protozoa, largely due to its high content of berberine, an isoquinoline alkaloid well recognized for its potent antiprotozoal activity (55). Beyond its direct antiparasitic action, berberine’s well established anti-inflammatory and immune modulating properties may also help strengthen the host’s immune defenses, amplifying its overall effectiveness as a treatment option (56).

#### 4.4.3 Nigella sativa

*Nigella sativa* (black seed), with both the seeds and their oil are rich in thymoquinone, a bioactive compound displaying antiparasitic and immunomodulatory properties (57, 58). Interestingly, *N. sativa* has demonstrated superior efficacy to nitazoxanide in mitigating *C. parvum* infection and restoring intestinal integrity. Its immunomodulatory effects, enhancing IFN-*γ* production and decreasing oocyst shedding; further highlight its promise as a supportive anti-cryptosporidial therapy (59). Research also reported that *N. sativa* may regulate immune functions, increasing the production of cytokines and other immune cells that can be crucial in combating infections (60).

#### 4.4.4 Turmeric (*Curcuma longa*)

Turmeric (*Curcuma longa*) has attracted considerable scientific interest for its possible role in treating infections caused by *Cryptosporidium*, mainly due to curcumin, its main bioactive ingredient. Curcumin possesses strong antiprotozoal, anti-inflammatory, and immunomodulatory properties (61, 62). Research has demonstrated that curcumin supplementation reshapes the gut microbiota composition, reduces *C. parvum* oocyst shedding, and prevents infection recurrence (61).

#### 4.4.5 Neem (*Azadirachta indica*)

Neem, a medicinal plant originating from South Asia, has demonstrated significant potential as a therapeutic agent for treating of protozoa due to its potent antiparasitic properties (63). Furthermore, *Azadirachta indica* (neem) demonstrates potent antiproliferative, antioxidant, and anti-inflammatory properties, with its leaf-derived flavonoid quercetin identified as an active compound effective against *C. parvum* (64).

Overall, turmeric (*Curcuma longa*) showed the strongest preclinical evidence against *Cryptosporidium*, with its component curcumin exhibiting antiparasitic and immunomodulatory properties. On the other hand, garlic (*Allium sativum*) inhibits parasite replication through allicin, while neem (*Azadirachta indica*) shows promising activity in preliminary studies. Turmeric remains the most promising candidate for clinical application, with garlic and neem requiring further validation for therapeutic strategies. In addition, despite promising preclinical evidence, most herbal compounds lack well-designed clinical trials in humans and animals.

## 5 Prophylactic measures, hygienic practices and public health interventions (environmental control)

### 5.1 Water treatment and sanitation

*Cryptosporidium* outbreaks have provided critical lessons for enhancing response and prevention frameworks. The 1993 Milwaukee incident, one of the largest documented waterborne outbreaks, underscored the necessity of implementing stringent water treatment protocols and continuous monitoring systems to interrupt transmission pathways (65). Similarly, outbreaks associated with recreational water venues have illuminated the crucial role of maintaining rigorous hygiene standards and elevating public education to mitigate infection risks (66). Agricultural environments further contribute to *Cryptosporidium* contamination, primarily due to the presence of fecal matter originating from both human and animal sources (67). However, *Cryptosporidium* oocysts can contaminate vegetables, fruits, juices, unpasteurized milk, raw meat, and fish through various sources such as infected food handlers, sewage contamination, agricultural runoff, and the use of manure from infected animals as fertilizer, potentially resulting in serious foodborne outbreaks (68). Among these sources, cattle manure represents a particularly significant risk, as an adult bovine can excrete over 36 million *C. parvum* oocysts daily. This observation highlights that manure from dairy or beef cattle operations can serve as a significant source of *C. parvum* contamination if effective manure management or treatment strategies are not implemented to reduce oocyst viability or prevent their transport to aquatic environments as recreational water sources (69). To address these risks, the mitigation of *Cryptosporidium* transmission through drinking water requires the implementation of scientifically validated treatment processes, including filtration, chlorination, ultraviolet irradiation, and ozonation. These interventions function to physically remove or chemically inactivate oocysts, thereby ensuring compliance with established microbiological water quality standards. In parallel, adherence to evidence-based hygiene practices, such as rigorous handwashing following defecation and prior to food handling, is essential for interrupting fecal-oral transmission pathways. Population-level health education and behavior change interventions serve as critical components of integrated control strategies, enhancing public awareness and promoting sustained adoption of preventive measures (70).

#### 5.1.1 Chemical treatments of water

Chemical disinfectants such as chlorine dioxide, ozone, and hydrogen peroxide play a pivotal role in controlling *Cryptosporidium* contamination, especially within water treatment processes. To further understand the critical roles of chemical disinfectants in controlling *Cryptosporidium*, the following section will examine the distinct properties and practical considerations associated with chlorine dioxide, ozone, and hydrogen peroxide in water treatment applications.

##### 5.1.1.1 Chlorine dioxide

Chlorine dioxide has emerged as a potent chemical agent for treating waterborne *Cryptosporidium* due to its strong oxidizing properties (71, 72). Recent investigations indicate that chlorine dioxide exhibits superior disinfectant efficacy compared with free chlorine in the inactivation of *Cryptosporidium* oocysts (73).

##### 5.1.1.2 Ozone

Ozone was the most effective treatment, achieving complete (100%) inactivation of *C. parvum* oocysts at a concentration of 24 mg/L through oxidation (74, 75). In addition, drinking water utilities that use ozone as a primary disinfectant and allow adequate contact time with a secondary disinfectant in clear wells or reservoirs can achieve substantially greater protection against *C. parvum* oocysts than with the primary disinfectant alone as chlorine (76).

##### 5.1.1.3 Hydrogen peroxide

Hydrogen peroxide has been widely studied as an antimicrobial compound targeting bacterial, fungal, and viral foodborne pathogens, owing to its potent oxidizing properties (77). Studies indicated that it reduces *Cryptosporidium* viability by damaging both cell membranes and internal structures (78). This oxidative stress leads to parasite inactivation and a marked decline in infective capacity (79–81). An environmental advantage of hydrogen peroxide is its ability to decompose into water and oxygen, leaving no harmful residues in treated environments. However, its effectiveness against *Cryptosporidium* can vary depending on factors such as concentration, contact time, and environmental conditions.

### 5.2 Personal hygiene

Rigorous personal hygiene is a fundamental measure for preventing the transmission of *Cryptosporidium*. Consistent and thorough handwashing with soap and clean water markedly reduces the risk of pathogen transfer through the fecal oral route. In childcare environments, thorough sanitation of toys and frequently touched surfaces is essential, particularly during outbreak periods. Individuals should avoid drinking untreated water or ice, especially in areas with questionable water safety, and should not swim during episodes of diarrhea or for at least 2 weeks after symptoms have fully resolved (82). Furthermore, caregivers who did not wash their hands after toileting were nearly three times more likely to have children infected with *Cryptosporidium* than handwashing ones, and those who relied on toilet paper alone were 1.6 times more likely to have infected children, demonstrating that toilet paper is not an effective hygiene measure unless combined with proper handwashing, safe waste disposal, and adequate sanitation (83).

Sustainable control requires combining individual hygienic practices with community level interventions that protect the microbiological quality of water supplies and break the transmission cycle of waterborne diseases (70). Therefore, cryptosporidiosis was highly prevalent among both cattle and their owners, with molecular detection of the *C. parvum* IId subtype in animal and human samples confirming zoonotic transmission. These findings, within a One Health framework, highlight the necessity for integrated surveillance and preventive strategies targeting livestock management, human health, and environmental hygiene to reduce the burden of this zoonotic infection (84).

## 6 Vaccination strategies

### 6.1 Current research on vaccines

Vaccine development targeting *Cryptosporidium* is progressing rapidly but remains complicated due to the parasite’s complex life cycle and antigen variation. To date, no vaccine with proven efficacy or favorable cost-effectiveness has been developed for *Cryptosporidium* (85). However various vaccine platforms have been explored, including development of recombinant protein vaccines as well as gp40- based vaccine, which have been primarily investigated in bovine models with the goal of activating both systemic and mucosal immunity (86, 87). Then, vaccination provided strong protection, as evidenced by a significant reduction in the severity and duration of diarrhea, improved calf health and weight gain, and the absence of mortality, with benefits observed in both suckling calves and calves that received colostrum from vaccinated dams (87). Despite promising preclinical results, clinical application is hindered by the parasite’s ability to evade immune detection and its complex interaction with host cells (1). Significant progress has been also made recently by identifying key antigens such as Cp15 and Cp23 that are essential for parasite survival and infection, allowing for more precise vaccine targeting (88). Numerous recent advances, including the use of DNA vaccines, novel vectors that stimulate mucosal immunity, and CpG oligonucleotides as adjuvants, may facilitate the development of vaccines (89).

It should be noted that the identification of immune targets and the development of effective vaccines are limited by the absence of a reliable *in vitro* system for parasite propagation. Consequently, reverse vaccinology approaches that use bioinformatic tools provide a compelling alternative for the discovery of vaccine candidates as a previously known vaccine candidate GP60 and a novel glycosylphosphatidylinositol (GPI) anchored antigens, CpH1 and CpSUB2 were confirmed as potential vaccine candidates against *C. parvum* infection (90). Moreover, a multi-epitope vaccine candidate designed from highly immunogenic epitopes may offer a promising and effective strategy for preventing *Cryptosporidium hominis* infection (91). Another recent study using multi-epitope subunit vaccine targeting immunogenic regions of *C. parvum* were performed in silico which provides a strong and promising basis for the development of safe and effective vaccines against cryptosporidiosis (92). Despite significant progress, fundamental biological and immunological challenges continue to impede *Cryptosporidium* vaccine development. Therefore, it is essential to dissect the parasite’s immune evasion mechanisms and address the complexities that must be overcome to achieve effective and sustained vaccine-induced protection. To provide a clear overview of the advancements and critical insights in *Cryptosporidium* vaccine research (Table 2).

**Table 2:** Implications from previous studies informing *Cryptosporidium* vaccine development.

Methodology	Research Approaches (*In vitro* /*In vivo*/Clinical)	Key Findings	References
Focused on selecting antigens that stimulate T-cell responses.	*In vitro*	Identified gp40/15 as a critical antigen, with IFN-*γ* as a key cytokine for immune defense against *Cryptosporidium*.	(89, 119)
Characterized sporozoite surface proteins (Cpgp40 and Cpgp40/15) and tested antibody-neutralization assays.	*In vitro*	Anti-gp40 and anti-gp40/15 antibodies significantly inhibited *Cryptosporidium parvum* infection.	(120)
Explored immunogenicity of gp40 and other surface antigens in animal and human models.	*In vivo*	gp40 induces T-cell activation, with promising immunogenic properties, though protective efficacy remains unclear.	(121, 122)
Investigated antigen-based vaccines, including profilin, P2 antigen, Muc4, and *Cryptosporidium* apyrase.	*Clinical/In vivo*	Highlighted the immunogenic potential of various antigens, though further validation is needed for practical application.	(123–125)
Evaluated CpGP15 recombinant antigen for *C. parvum* infection elimination in cattle.	Clinical	Demonstrated CpGP15’s effectiveness in clearing infections and improving diagnostic accuracy in animal farms.	(126)
Studied CP15 and circumsporozoite-like (CSL) peptides in vitro.	*In vitro*	CP15 and CSL peptides stimulated antibody production and neutralized parasite entry into host cells.	(86)
Investigated thrombospondin-related anonymous protein (TRAP) in *C. parvum* and other apicomplexans.	*In vitro/In vivo*	TRAP proteins facilitate gliding motility and host-cell invasion, marking them as strong candidates for vaccine development.	(127)
Studied TRAP-C1, a protein located at the apical end of *Cryptosporidium* sporozoites.	*In vitro*	TRAP-C1 is essential for cell penetration and gliding motility, supporting its potential as a vaccine candidate.	(128, 129)
Examined CpTSP8, a TRAP-like protein located in sporozoites and merozoites.	*In vitro/In vivo*	CpTSP8 is crucial for gliding motility, invasion, and attachment to host cells, making it a promising target for vaccine development.	(130, 131)
Characterized Cp-P34, a novel sporozoite surface protein, in naturally exposed alpacas.	*In vivo*	Cp-P34 stimulates immune responses and appears transiently on the sporozoite surface, suggesting its role in gliding and host-cell attachment processes.	(132)

### 6.2 Challenges in vaccine development

The pursuit of an effective vaccine against *Cryptosporidium* is hindered by the parasite’s intricate biology and sophisticated strategies for immune evasion. Its complex lifecycle, which encompasses both asexual and sexual phases, complicates the identification of optimal antigenic targets for vaccine development. Although the immune system contributes to the control of established *C. parvum* infection, immunity acquired following primary exposure does not necessarily provide protection against reinfection. As far, active vaccination of newborn livestock faces significant practical and immunological challenges, whereas passive immunization; achieved by immunizing dams to provide protective antibodies to their offspring through colostrum, has emerged as a valuable and effective alternative strategy (93). The lack of reliable animal models limits preclinical evaluation of vaccine candidates. Moreover, since the parasite colonizes intestinal epithelial cells within the gastrointestinal tract, eliciting a strong and lasting mucosal immune response remains a major challenge. A controlled human infection model (CHIM) for *Cryptosporidium* in healthy adult volunteers provides a robust clinical proof-of-concept platform for evaluating novel therapeutics and vaccine as well as this model has the potential to accelerate the development of both drugs and vaccines for cryptosporidiosis (94).

## 7 Challenges in treatment and prophylaxis

### 7.1 Exceptional biological traits of *Cryptosporidium*

*Cryptosporidium* possesses unique biological features that complicate drug discovery, including its intracellular but extracytoplasmic niche, where it is separated from the host cytoplasm by an electron-dense band as the host plasma membrane folds up to envelop the invading sporozoite and forms the parasitophorous vacuole membrane, poses significant challenges for antiparasitic drug discovery (95). These characteristics restrict the effectiveness of conventional antiparasitic screening approaches and contribute to poor compound selectivity. Many candidate compounds demonstrated efficacy *in vitro* systems or *in vivo* models but failed to translate to clinical trials, which more accurately reflect severe human disease. This limitation has hindered the progression of promising leads into clinical development (30).

### 7.2 Drug resistance

The emergence of drug-resistant *Cryptosporidium* strains poses a significant challenge to the effective management and control of cryptosporidiosis. Hasan et al. (96) evaluated the highly potent *C. parvum* methionyl-tRNA (CpMetRS) synthetase inhibitor 2093, using a neonatal calf model of cryptosporidiosis. In experimentally infected dairy calves, the administration of compound 2093 led to an initial decrease in oocyst shedding during the first 4 days post-infection. However, by day five, parasite shedding resumed in most treated calves, suggesting the probable emergence of drug resistance. This resistance is attributed to mutations in CpMetRS that resulted in amino acid substitutions. The rapid development of resistance was likely facilitated by the high parasite burden in the dairy calf model; however, it remains uncertain whether additional tolerance or resistance mechanisms contribute to parasite persistence.

On other hand, to distinguish whether anti-cryptosporidial drug activity targets the parasite or the host cell, a previous study (97) developed an advanced *in vitro* model using stable MDR1-transgenic HCT-8 cells with enhanced drug tolerance. Nitazoxanide, the frontline treatment for cryptosporidiosis, has limited efficacy, particularly in immunocompromised patients. While, alternative therapies are also suboptimal, complicating clinical management and highlighting the need for new treatment strategies (1).

### 7.3 Variability in response to treatment

The management of *Cryptosporidium* infections remains complex due to the heterogeneous therapeutic outcomes observed among different patient groups. In immunocompetent hosts, the infection often manifests as a self-limiting diarrheal illness that may be resolved without targeted treatment. In contrast, immunosuppressed individuals, including those living with HIV/AIDS, are prone to severe, persistent infections necessitating active medical management (1). The variability in clinical response is shaped by multiple factors, including the host’s immune competence, specific *Cryptosporidium* species and genotypes involved (98).

### 7.4 Current limitations in the development of conventional and herbal remedies for *Cryptosporidium*

The development of novel pharmacological and herbal treatments for *Cryptosporidium* infection faces substantial challenges due to the parasite’s unique biology and the complex determinants of therapeutic efficacy. A primary barrier in drug discovery is the absence of reliable *in vitro* and *in vivo* models that faithfully replicate the complexities of human infection, thereby limiting the accurate assessment and high-throughput screening of candidate compounds. Furthermore, the parasite’s capacity to develop resistance against frontline drugs such as nitazoxanide, coupled with difficulties in delivering effective drug concentrations to the intestinal epithelium, significantly impedes successful treatment outcomes (33).

### 7.5 Culturing *Cryptosporidium* oocysts

Establishing reliable *in vitro* culture systems for *Cryptosporidium* oocysts remains a major obstacle, hindering progress in parasite biology and therapeutic development. The complex life cycle of *Cryptosporidium*, requiring specific host cells and environments to develop and proliferate properly (99). Unlike many other pathogens, long term culture remains constrained by persistent limitations, frequently necessitating reliance on animal models or specialized cell lines that are limited in availability and impractical for large-scale experimentation. Additionally, maintaining continuous Cryptosporidium infection in vitro culture systems can be problematic due to their strict environmental requirements for temperature, pH, and nutrient composition (99, 100). These challenges significantly constrain high-throughput drug screening and in-depth investigations into parasite pathogenesis.

### 7.6 Socioeconomic determinants

The successful implementation of prophylactic measures against *Cryptosporidium* infections is strongly influenced by socioeconomic factors, particularly in low-resource settings. Limited access to safe drinking water and inadequate sanitation infrastructure substantially increase the risk of waterborne transmission (101). Among Zambian children with HIV-related immunosuppression, nitazoxanide, despite higher doses and prolonged treatment, did not eliminate cryptosporidiosis or alleviate symptoms which confirmed the drug’s lack of efficacy in this patient group (31). Addressing these barriers requires integrated approaches that consider socioeconomic disparities, emphasizing the need for improved infrastructure, community education, and affordable healthcare solutions to effectively mitigate the burden of *Cryptosporidium* infections in resource limited settings.

## 8 Advances in diagnostics

Microscopic analysis of fecal samples continues to be a key method for diagnosing *Cryptosporidium* infection, utilizing the parasite’s distinctive oocyst features as well as staining properties, for identification (102). Another approach is antigen detection assays, including enzyme immunoassays (EIAs) and rapid diagnostic tests, which detect specific *Cryptosporidium* antigens in stool samples, providing rapid results suitable for point-of-care settings (98). Molecular methods, such as polymerase chain reaction (PCR) and nucleic acid amplification techniques, offer high specificity and sensitivity by detecting parasite genetic material which also enable precise species identification and genotyping, which are essential for detailed epidemiological analysis and outbreak investigations (103). Future progress in diagnosing *Cryptosporidium* infections is expected through the use of next-generation sequencing on stool samples, which enables the simultaneous detection of multiple pathogens and their genetic variants as well as molecular diagnostic techniques are likely to become more widely adopted, though their integration into clinical laboratories will require substantial adjustments to existing workflows (98). Furthermore, emerging point of care testing (POCT) platforms utilize molecular methods such as loop mediated isothermal amplification (LAMP) and nucleic acid amplification tests (NAATs) to identify *Cryptosporidium* DNA with high accuracy and sensitivity outside traditional laboratories (104).

## 9 Implementation of treatment and prophylactic measures in various settings

The adoption of therapeutic and preventive strategies for *Cryptosporidium* varies markedly between regions, shaped by disparities in healthcare systems, socioeconomic conditions, and environmental context. In resource-limited settings, treatment and prevention are often constrained by inadequate healthcare access and the high expense of antiparasitic agents such as nitazoxanide, limiting both therapeutic reach and prophylactic coverage (31). Furthermore, the anthroponotic subtype *C. parvum* IIc is predominantly found in low-income regions with poor sanitation and among HIV-positive individuals, while it is rarely observed in high-income countries. Considering the significant burden of cryptosporidiosis in low-resource settings and the pathogenic potential of related human-specific species such as *C. hominis*, *C. parvum* IIc is emerging as a key contributor to the disease (101).

## 10 Future directions and research priorities

### 10.1 Areas requiring further research

The development of novel therapeutic agents against *Cryptosporidium* remains a critical priority, with special emphasis on discovering innovative chemical entities and identifying unexplored molecular targets to counteract the emergence of drug-resistant strains and enhance clinical efficacy (33, 96). Research focused on phytochemical and synthetic compounds continues to be essential, requiring rigorous standardization of formulations, comprehensive evaluation of therapeutic potential (33). In parallel, vaccine development constitutes a major research frontier, aiming to elicit long-lasting protective immunity, particularly in immunocompromised and other high-risk populations (105). Progress in this domain must be accompanied by the refinement of diagnostic technologies, with a focus on producing rapid, ultra-sensitive, and field-adaptable assays capable of detecting *Cryptosporidium* with high specificity.

### 10.2 Potential impact of new technologies

#### 10.2.1 Genomic and proteomic approaches

Recent genomic and proteomic advancements hold significant potential to enhance the understanding, diagnosis, and treatment of *Cryptosporidium* infections. Techniques like whole-genome sequencing and comparative genomics enable detailed characterization of species and strains, paving the way for improved disease management (106, 107). These methods reveal important insights into genetic diversity, population structure, and host–parasite interactions, which are crucial for improving molecular epidemiology and informing targeted public health interventions. Proteomic studies further characterize protein interactions, functions, and cellular pathways, supporting the identification of potential diagnostic biomarkers and informing the development of novel therapeutic strategies (106). In parallel, proteomic studies provide essential information about the full range of proteins expressed by *Cryptosporidium* during its different life stages. Using techniques such as mass spectrometry, critical proteins have identified which play roles in host invasion, metabolism, and immune evasion (106). This knowledge is vital for elucidating how the parasite causes disease and interacts with its host, guiding the development of more targeted therapies. Combined genomic and proteomic approaches can significantly enhance diagnostic accuracy, as genomic tests enable the highly sensitive detection of specific *Cryptosporidium* DNA sequences, facilitating early diagnosis and precise species identification (108). Meanwhile, proteomic profiling helps uncover new protein markers that can increase the accuracy of antigen tests and rapid detection kits. These technologies also strengthen public health monitoring and outbreak response. Additionally, genomic data help trace transmission pathways, identify sources of outbreaks, and reveal how different strains are related, all of which are key for controlling the spread of infection. However, large genomic datasets require advanced bioinformatics tools and skilled analysis. Meanwhile, using CRISPR/Cas9 in *Cryptosporidium* has allowed researchers to create reporter strains and directly study the function of parasite genes, opening new opportunities for drug discovery (8).

#### 10.2.2 Artificial intelligence in drug discovery

Artificial intelligence (AI) has become a powerful catalyst in advancing both pharmaceutical research and the study of disease dynamics by utilizing sophisticated computational methods to expedite the discovery of new drugs and enhance disease monitoring systems. In drug development, AI enhances the prediction of molecular interactions and the optimization of chemical compounds, accelerating the discovery of effective therapies candidates beyond the limits of traditional approaches (109). Based on a study employing computational structure and function predictions, the identified *C. hominis* hypothetical protein (TU502HP) was proposed as a promising molecular target for the design of an inhibitory molecule (110). Through comprehensive analysis of extensive genomic and proteomic datasets, machine learning uncovers intricate biological patterns that inform the creation of precise therapies for cryptosporidiosis. In epidemiological investigations, explainable AI supports the real-time processing of diverse data streams and builds predictive frameworks to anticipate disease trends, recognize outbreak signals, and aid strategic public health decision-making (111). Moreover, AI might accelerate genomic investigations by swiftly examining large volumes of *Cryptosporidium* genetic material, pinpointing mutations linked to increased pathogenicity, resistance to treatments, and transmission characteristics. A deeper understanding of parasite biology enables the development of more precise and effective disease management strategies. However, widespread implementation of AI in this field faces challenges such as ensuring data quality, promoting algorithmic transparency, and addressing ethical concerns related to privacy and bias. Overcoming these obstacles will require interdisciplinary collaboration, robust regulatory frameworks, and ongoing innovation in AI technologies (112, 113).

## 11 Policy recommendations and global health strategies

Effective global health policies are essential to confront the ongoing challenge posed by *Cryptosporidium* infections and to improve health outcomes on a worldwide scale. Key policy priorities include enhancing disease monitoring systems to accurately assess infection dynamics, implementing strict water sanitation and hygiene standards to interrupt transmission pathways, and increasing public education to promote preventive measures and treatment awareness. International partnerships in terms of One Health approach is critical to establish unified diagnostic standards, facilitate the exchange of epidemiological information, and allocate resources efficiently to regions with high disease burden (114). Additionally, fostering innovation through support for research in therapeutics, vaccine development, and diagnostic advancement is fundamental. Integrating these elements into global health strategies strengthens resilience against *Cryptosporidium*, ultimately advancing public health security and reducing disease incidence across diverse populations.

## 12 Conclusion

Given the comprehensive examination of *Cryptosporidium* treatment, prophylaxis, diagnostics, and global health strategies, addressing the multifaceted challenges of *Cryptosporidium* infection demands a comprehensive One Health perspective that integrates human, animal, and environmental health. Progress in this field depends on expanding therapeutic options, including agents like nitazoxanide, paromomycin, and azithromycin, alongside continued research into natural compounds and novel chemical candidates, each offering distinct advantages and limitations. Enhancing diagnostic capabilities, advancing vaccine development, and applying artificial intelligence to monitor disease trends all represent valuable directions for future research and public health practice. Overcoming the burden of cryptosporidiosis also calls for strong policies, improved surveillance systems, safe water infrastructure, and effective public education to limit transmission and outbreaks. Cross-disciplinary collaboration and international cooperation remain essential to strengthen treatment success, prevent widespread infection, and reduce the overall impact of this parasite. By combining innovation, evidence-based strategies, and shared global commitment, meaningful strides can be achieved in protecting vulnerable populations and improving public health worldwide.
